# Statistical learning across passive listening adjusts perceptual weights of speech input dimensions

**DOI:** 10.1016/j.cognition.2023.105473

**Published:** 2023-05-19

**Authors:** Alana J. Hodson, Barbara G. Shinn-Cunningham, Lori L. Holt

**Affiliations:** a Department of Psychology, Carnegie Mellon University, Pittsburgh, PA, USA; b Neuroscience Institute, Carnegie Mellon University, Pittsburgh, PA, USA

**Keywords:** Statistical learning, Perceptual weight, Speech categorization, Dimension-based statistical learning

## Abstract

Statistical learning across passive exposure has been theoretically situated with unsupervised learning. However, when input statistics accumulate over established representations – like speech syllables, for example – there is the possibility that *prediction* derived from activation of rich, existing representations may support error-driven learning. Here, across five experiments, we present evidence for error-driven learning across passive speech listening. Young adults passively listened to a string of eight *beer* - *pier* speech tokens with distributional regularities following either a canonical American-English acoustic dimension correlation or a correlation reversed to create an accent. A sequence-final test stimulus assayed the perceptual weight – the effectiveness – of the secondary dimension in signaling category membership as a function of preceding sequence regularities. Perceptual weight flexibly adjusted according to the passively experienced regularities even when the preceding regularities shifted on a trial-by-trial basis. The findings align with a theoretical view that activation of established internal representations can support learning across statistical regularities via error-driven learning. At the broadest level, this suggests that not all statistical learning need be unsupervised. Moreover, these findings help to account for how cognitive systems may accommodate competing demands for flexibility and stability: instead of overwriting existing representations when short-term input distributions depart from the norms, the mapping from input to category representations may be dynamically – and rapidly – adjusted via error-driven learning from predictions derived from internal representations.

## 1. Introduction

The world presents considerable variability, but there is also structure. The regularities that lurk within perceptual input have an important impact on behavior. Speech recognition, for instance, is influenced by transitional probabilities across syllables (Saffran, Aslin, and Newport, 1996; Saffran and Wilson, 2003; Thiessen and Saffran, 2003), patterns of correlated acoustic features (Idemaru and Holt, 2011; Liu and Holt, 2015; Maye, Weiss, and Aslin, 2008), and the mean of long-term average spectra (Daikoku, Yatomi, and Yumoto, 2014; Holt, 2005). Each of these examples demonstrates sensitivity to stimulus statistics accumulated over time, and a corresponding influence on subsequent behavior. But it is unclear whether a common process underlies these, and other, cases of detecting and exploiting input regularities.

Over the past decades, understanding how humans make use of statistical input regularities has developed into the vital and productive enterprise of understanding *statistical learning* (Armstrong, Frost, and Christiansen, 2017; Aslin, 2017; Frost, Armstrong, and Christiansen, 2019; Saffran and Kirkham, 2018; Sherman, Graves, and Turk-Browne, 2020). In large part, this research has examined how human infants, children, and adults detect and utilize input regularities in an unsupervised fashion across passive exposure, implicitly and without behavioral response (Fiser and Aslin, 2002; Saffran et al., 1996; Turk-Browne, Scholl, Johnson, and Chun, 2010). Nevertheless, even infant learners are not blank slates. Familiar input – like native speech – activates existing representations. In doing so, it may generate predictions built from long-term statistical norms. Prior research examining overt speech categorization demonstrates that when speech input regularities violate these predictions, there is rapid, online learning to adjust the informativeness of acoustic input dimensions in speech categorization (Idemaru and Holt, 2011, 2014, 2020; Liu and Holt, 2015; Wu and Holt, 2022; Zhang and Holt, 2018). This suggests that predictions generated from existing representations might support error-driven learning in cases in which input regularities depart from expectations (Knudsen, 1994). This learning is *statistical* in the sense that it is driven by input regularities, but it is not *unsupervised* in the sense traditionally ascribed to statistical learning.

However, we do not yet know if error-driven learning in speech is dependent upon active categorization and overt decisions, or if instead the influence of these regularities can accumulate over passive listening. Here, across five studies, we capitalize on a case of rapid, online learning across speech input regularities for which there is evidence for error-driven learning (Wu and Holt, 2022; Zhang, Wu, and Holt, 2021). We ask whether this learning depends upon overt categorization decisions and responses or if passive exposure to speech input regularities may be sufficient.

### 1.1. Dimension-based statistical learning

An emerging literature has demonstrated robust and replicable effects of short-term distributional regularities in modulating how strongly a particular acoustic dimension influences perceived speech category identity, its *perceptual weight* (Holt and Lotto, 2006; Idemaru and Holt, 2011; Lehet and Holt, 2020; Schertz, Cho, Lotto, and Warner, 2016). Typically, no single acoustic dimension is necessary or sufficient to define speech category membership. Rather, multiple dimensions covary and differ in the effectiveness with which they signal a category. As an example, both voice-onset-time (VOT, the time that elapses between the release of a consonant and the start of voicing from vibration of the vocal folds) and fundamental frequency (F0, the frequency of vibration of this voicing) differentiate /b/ from /p/ in American English (Abramson and Lisker, 1985; Castleman and Diehl, 1996; Kohler, 1985; Whalen, Abramson, Lisker, and Mody, 1993). Moreover, VOT and F0 covary in a particular manner: shorter VOTs and lower-frequency F0s typically signal /b/, whereas longer VOTs and higher-frequency F0s signal /p/. Additionally, although both acoustic dimensions contribute to /b/–/p/ categorization, VOT is more diagnostic than F0; it carries greater perceptual weight (Francis, Kaganovich, and Driscoll-Huber, 2008; Lisker, 1986; Wu and Holt, 2022; Yu, 2022).

These baseline perceptual patterns reflect American English speech input (Kingston and Diehl, 1994). Yet, short-term input sometimes violates these norms. We might encounter a stranger with an unfamiliar dialect, or a spouse with a head cold. Systematic shifts in speech input like this negatively impact comprehension (Bradlow & Bent, 2008; Clarke & Garrett, 2004). Yet, a bit of experience with such speech can be sufficient for comprehension to improve, and even for improvements to generalize to other contexts or talkers with similar acoustic shifts (Bradlow & Bent, 2008; Davis, Johnsrude, Hervais-Adelman, Taylor, and McGettigan, 2005; Samuel & Kraljic, 2009). There is no clear consensus on what drives these adjustments, but one line of research has shown that listeners track distributional speech regularities and that perceptual weights of acoustic dimensions adjust when short-term inputs mismatch long-term norms (Hodson, DiNino, Shinn-Cunningham, and Holt, 2022; Idemaru and Holt, 2011, 2014, 2020; Idemaru and Vaughn, 2020; Jasmin, Tierney, Obasih, and Holt, 2021; Lehet and Holt, 2017, 2020; Liu and Holt, 2015; Schertz, Cho, Lotto, and Warner, 2015; Wu and Holt, 2022; Zhang et al., 2021; Zhang and Holt, 2018).

As an example, consider what happens when speech input shifts so that the typical English VOTxF0 correlation flips, with /b/ now associated with lower-frequency F0s and /p/ with higher-frequency F0s in an ‘artificial accent.’ Introduction of an accent with short-term speech input statistics that deviate from the language-community norm produces rapid perceptual adjustments (Idemaru and Holt, 2011, 2014). Specifically, listeners rapidly down-weight reliance on F0 in speech categorization decisions, so that F0 is even less effective in signaling /b/–/p/ categories.

This pattern of *dimension-based statistical learning* has been observed for both consonants (Idemaru and Holt, 2011, 2014, 2020; Idemaru and Vaughn, 2020; Schertz, Kang, and Han, 2019; Wu and Holt, 2022; Zhang et al., 2021; Zhang and Holt, 2018) and vowels (Lehet and Holt, 2020; Liu and Holt, 2015; Wu and Holt, 2022), as well as for a suprasegmental contrast (Jasmin et al., 2021). For each instance, listeners exhibit exquisite sensitivity to evolving statistical regularities across the multiple acoustic input dimensions that signal speech categories; the mapping from speech input to categories is flexible, not fixed. Usefully, dimension-based statistical learning provides a means of assaying exactly *how* this sensitivity affects the mapping; reliance on a secondary dimension, like F0, is down-weighted.

These effects are unambiguously *statistical* learning in that that listeners exhibit sensitivity to the evolving short-term regularities in speech input. Yet, all experiments to date have required listeners to make overt categorization decisions to individual utterances, with short-term speech input statistics accumulating across trials (and responses). No one has tested if listeners accumulate short-term dimension regularities across passive listening and, if they do, whether passive exposure can drive down-weighting of the secondary dimension, as observed for regularities accumulated across overt categorization decisions.

This question is especially relevant because dimension-based statistical learning has been proposed to arise from error-driven learning (Idemaru and Holt, 2011; Liu and Holt, 2015; Wu and Holt, 2022; Zhang et al., 2021). By this view, information that disambiguates systematic shifts in speech acoustics – whether lexical (Davis et al., 2005; Norris, McQueen, and Cutler, 2003; Schwab, Nusbaum, and Pisoni, 1985), visual information from articulating faces (Bertelson, Vroomen, and De Gelder, 2003; Vroomen, van Linden, de Gelder, and Bertelson, 2007), orthographic feedback (Guediche, Fiez, and Holt, 2016; Schwab et al., 1985), or unambiguous acoustic speech cues like VOT (as in the example discussed above; Idemaru and Holt, 2011) – resolves the mapping of ambiguous speech acoustics to a particular speech category. In doing so, these mappings may generate expectations of category-typical speech input. If the acoustic input is a poor match to predictions, an error signal may shift speech categorization – an effect that persists even when the disambiguating information is no longer present (see Guediche, Blumstein, Fiez, and Holt, 2014 for a review of this perspective). In this way, and convergent with other demonstrations of speech adaptation to adverse listening conditions, the mapping of acoustics to speech categories may be driven by supervisory teaching signals available from disambiguating information sources, which drive activation of existing representations (Bertelson et al., 2003; Guediche et al., 2014, 2016; Idemaru and Holt, 2011; Norris et al., 2003).

Based on this logic, Wu and Holt (2022) posited that categorization accuracy (as defined by the primary dimension) serves as a behavioral index of successful category activation. Correspondingly, by the reasoning outlined above, categorization accuracy upon introduction of an accent should relate to the magnitude of the down-weighting of the secondary dimension. Moreover, when a change in listening context (e. g., speech-in-noise) prompts a shift in perceptual weights – such that a secondary dimension becomes primary in signaling category identity – the new primary dimension will drive category activation. Consequently, in this context, the formerly primary (now secondary) dimension will be down-weighted upon introduction of the accent. Wu and Holt report evidence to support each of these predictions of the error-driven model, across both consonant and vowel categorization.

As additional evidence, top-down resolution of speech category activation via lexical knowledge alone can drive dimension-based statistical learning (Zhang et al., 2021). Ordinarily VOT would play a strong role in signaling /b/–/p/ category identity, but Zhang and colleagues held VOT constant at a perceptually ambiguous value. They reasoned that if top-down feedback from lexical representations were sufficient to activate /b/–/p/ categories differentially then it may drive dimension-based statistical learning even in the absence of bottom-up acoustic regularities. For example, presenting a high F0, VOT-ambiguous sound in the context of *__eef* encourages perceptual resolution of the stimulus as /b/ (*beef* is a word, *peef* is not) but the same sound is more often heard as /p/ in the context of *__eace* (*peace* is a word, *beace* is not). In this way, Zhang and colleagues conveyed ‘phantom’ short-term stimulus statistics that do not exist in the input: for instance, a VOT-ambiguous, low-F0 stimulus paired with *__eef* accords with canonical English VOT-F0 regularities whereas paired with __*eace* it conveys the opposite VOT-F0 regularity. Phantom distributions reliant on top-down category activation produced dimension-based statistical learning. In summary, dimension-based statistical learning depends upon a ‘teacher signal’ that drives category activation via bottom-up (e. g., an unambiguous VOT) or top-down (e.g., *beef* is a word, *peef* is not; Ganong, 1980) resolution, a proposition consistent with error-driven learning.

Yet, all prior studies of dimension-based statistical learning have employed tasks with an explicit categorization decision and overt response on each trial. Is passive exposure to short-term distributional regularities sufficient to produce perceptual down-weighting? Or are predictions that drive dimension-based statistical learning dependent on category activation that arises from explicit category decisions and responses? Answering these questions will be critical in understanding interactions of error-driven learning via prediction and statistical learning, and in determining whether dimension-based statistical learning – whatever its origins – plays a role in more natural listening contexts that do not demand explicit category decisions.

Experiment 1 establishes a novel approach across three replications. We present statistically structured sequences of speech syllables across passive listening, then prompt listeners to categorize F0-differentiated test stimuli to assess perceptual weight as a function of the statistical input accumulated over passive exposure. Experiment 2 directly compares the magnitude of learning in the overt category decision paradigm used in prior literature to the novel passive listening paradigm using a within-subjects design. Finally, Experiment 3 examines the robustness and rapidity of these effects by examining whether learning is influenced by exposure to short-term regularities that are blocked, versus mixed, across trials.

### 1.2. Analysis plan

The dependent measure was a two-alternative (*beer-pier*) speech categorization of the F0-differentiated test stimuli, so we submitted results to a general linear mixed effects model with a binomial linking function (logistic regression), using the *glmer* function from the lme4 R package.

We began with construction of empty models (no fixed effects) to determine the random effects structure, and iteratively added effects until obtaining the maximal structure. The maximal structure contained a random slope of the Block (Canonical, Reverse) and Test Stimulus (High F0, Low F0) interaction that varied by a random intercept of Subject. However, in some instances the data could not support the maximal structure, resulting in a singular fit. In these cases, we reduced the random effects structure to a random slope of Test Stimulus that varied by Subject. These modifications are noted in Table 3.

We next used the R *anova* function to compare different random effects structures to the empty model. The chi-square statistic (from the likelihood-ratio test) determined whether models differed significantly in their fit; the AIC and BIC statistics quantified the quality of fit. We used the best random effects structure for the fixed effects models, following the same iterative process of adding effects and their interaction terms, and then conducting model comparison.

All models included the fixed effects of Block, Test Stimulus, and the Block x Test Stimulus interaction. This interaction term is the critical effect of interest as it represents the dimension-based statistical learning effect. The reference level for Block was always “canonical”, and the reference for Test Stimulus was always “Low F0”. Experiments 1c and 2 involved an additional within-subjects factor, described in their respective sections.

All fully interactive model outputs and parameters are included in Tables 2 and 3.

## 2. Experiment 1

Experiment 1 examines whether passive exposure to short-term distributional regularities evolving across a sequence of speech syllables elicits dimension-based statistical learning. Experiment 1a offers concurrent visual support (clipart displays) across the passively experienced, statistically structured speech sequence to encourage unambiguous speech category activation across passive listening. Experiment 1b eliminates this visual crutch. Finally, Experiment 1c blends these two approaches in a within-subjects design to interrogate the potential influence of category support from visual images. This final experiment also provides insight into the time course of learning, as each task is half as long to maintain total study length across experiments.

### 2.1. Methods

#### 2.1.1. Participants

Previous studies have examined dimension-based statistical learning elicited within tasks in which participants make an overt categorization response on each trial, across both exposure and test stimuli and among in-person participants. These studies can provide rough guide for power estimates, but because Experiment 1 introduces a novel paradigm we do not yet have an appropriate effect size estimate. Guided by one such prior study (Zhang and Holt, 2018) we would anticipate an effect size for the crucial Block x Test Stimulus interaction to be approximately 0.44, which would require 16 participants to achieve power of at least 0.80 at a significance level of 0.05 (see Zhang and Holt, 2018 for details). Therefore, guided by this imperfect estimate we oversampled (*N* = 30 per study) to ensure that we could detect subtle effects in this new paradigm conducted with online participants.

Across all studies, younger adults (18–31 years) were recruited via Prolific (www.prolific.sc) and tested online using the Gorilla Experiment Builder (www.gorilla.sc). All participants were native speakers of American English with self-reported normal hearing. The experiment included control trials that were unambiguous exemplars of /b/ and /p/ (see below); data from subjects achieving <70% accuracy on control trials were excluded in analyses to protect against inattentive online participants. Table 1 presents participant demographics across studies; individuals participated in at most one study. Informed consent was obtained in compliance with a protocol approved by Carnegie Mellon’s Institutional Review Board.

#### 2.1.2. Stimuli

Stimuli were based on Idemaru and Holt (2011). Using a cross-splicing procedure (McMurray, Tanenhaus, & Aslin, 2002), we generated 7 VOT steps (0 ms to 30 ms, 5-ms steps) from natural utterances of a female monolingual English speaker saying *beer* and *pier*. Using Praat 5.0 (Boersma & Weenink, 2010), we manipulated the onset fundamental frequency (F0) to create 7 F0 levels (200–320 Hz, 20-Hz steps) for each VOT. This onset F0 frequency was maintained for 80 ms, then decreased linearly over 150 ms to 180 Hz. This created a 2-dimensional grid of speech exemplars varying perceptually from *beer* to *pier* across variation in VOT and F0 acoustics. Each stimulus was encoded as a .FLAC sound file and normalized to the same root-mean-square (RMS) amplitude.

**Fig. 1.**
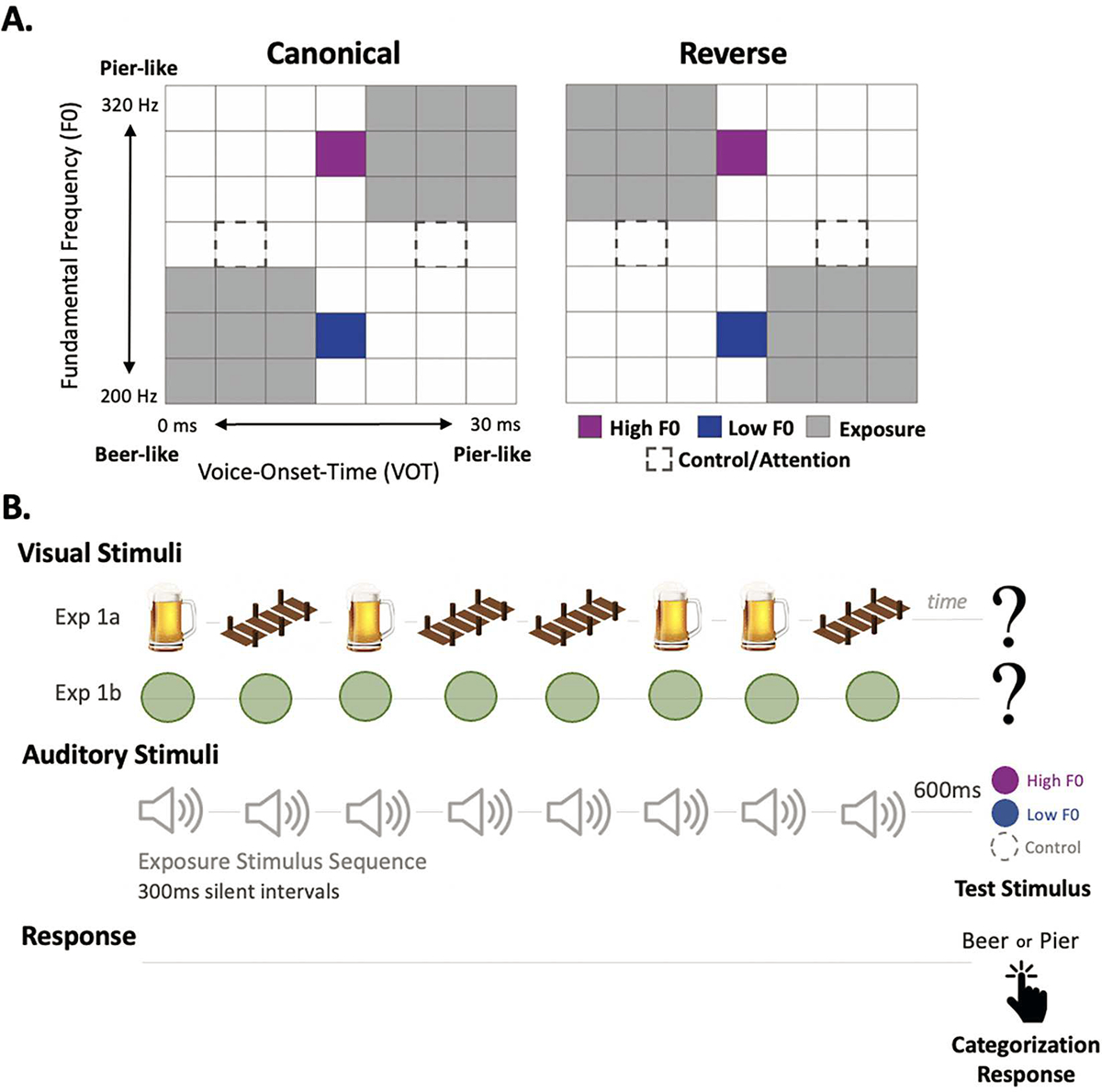
Stimulus schematic and Task Design. *A*, Voice onset time and fundamental frequency vary parametrically to create an acoustic space that ranges perceptually from *beer* to *pier*. Exposure stimuli (grey) convey a short-term distribution in speech input. Test stimuli (purple, blue) are constant across blocks and assess participants’ reliance on F0 in categorization. Control stimuli (dashed line) serve as an attention check among online participants. *Left*, Canonical VOTxF0 regularity consistent with English. *Right*, Reverse VOTxF0 regularity conveying an ‘accent.’ *B*, Task design for Exp1a (beer/pier clipart), Exp1b (green circles), and Exp1c (within-subjects comparison of Exp 1a and 1b). *Top*, visual stimuli are displayed in synchrony with the onset of each auditory stimulus. A black question mark indicates the test stimulus. *Middle*, auditory stimuli were presented in a string of eight Exposure stimuli (4 *beer,* 4 *pier;* random order, 300 ms ISI) followed after 600 ms by one Test stimulus (HighF0, LowF0, or Control). *Bottom*, participants listen passively to Exposure stimuli and make a categorization decision (“beer” or “pier” via keyboard press) based on the Test stimulus.

Fig. 1 illustrates the distributions of stimuli sampled from this 49-token two-dimensional acoustic space. Exposure stimuli conveyed the short-term speech input regularity (grey, Fig. 1). Since native English listeners perceptually weight VOT more than F0 (Francis et al., 2008; Lisker, 1986; Wu and Holt, 2022; Yu, 2022), the perceptually unambiguous VOT of Exposure stimuli strongly signaled category identity across blocks. Canonical block exposure stimuli mirroring English speech regularities (Fig. 1A, left) involved nine exemplars of *beer* with short VOT (0–10 ms) and low F0 (200–240 Hz) and 9 exemplars of *pier* with long VOT (20–30 ms) and high F0 (280–320 Hz). In contrast, Reverse block exposure stimuli involved exemplars with the opposite VOTxF0 correlation (Fig. 1A, right) across Exposure stimuli.

We assessed the influence of these short-term regularities on the perceptual weight of F0 with two Test stimuli held constant across blocks and defined by a perceptually ambiguous VOT (15 ms) that demanded reliance on the Low F0 (220 Hz) or High F0 (300 Hz) to signal category identity. Two additional Control stimuli with perceptually unambiguous VOT (5 ms, 25 ms) and an intermediate F0 (260 Hz) assessed online participants’ task engagement. Participants who did not categorize these unambiguous stimuli with 70% accuracy were excluded from analyses.

#### 2.1.3. Procedure

Participants in each experiment completed the study online using the Chrome internet browser on their own laptop or desktop computer (no smartphone or tablet) and their own headphones; operating system was not restricted. Participants received a reminder to turn off computer notifications and completed a brief headphone and sound-level check (Milne et al., 2021).

Each of the 96 approximately 7-s trials was composed of eight Exposure stimuli followed by a Test stimulus. Participants passively listened to Exposure stimuli and overtly labeled the final, Test stimulus as *beer* or *pier*. Each sequence of eight Exposure stimuli included four random selections of *beer* (VOT <15 ms) and 4 random selections of *pier* (VOT > 15 ms), presented in a random order with 300 ms inter-stimulus silent intervals. A 600 ms silent interval followed each sequence, after which a Test (or Control) stimulus was presented, accompanied by a black question mark. At this prompt, participants responded “beer” or “pier” by pressing the F or J key, respectively (Fig. 1c).

All participants first experienced sequences sampling the Canonical VOTxF0 regularity (Fig. 1a) consistent with the long-term norms of English across 48 trials. A second 48-trial block conveyed a the Reverse VOTxF0 ‘accent’ (Fig. 1b). Self-timed breaks were offered halfway through each block and between blocks.

Experiments 1a and 1c provided additional category support by presenting a clip art image of either a *beer* or a *pier* with each Exposure stimulus. Stimuli with a VOT <15 ms were accompanied by a *beer* image; those >15 ms were accompanied by a *pier*. In experiments without additional category support (Exp 1b, 1c), a progress bar of green circles filled in at the onset of each sound.

Test and Control stimuli, to which responses were made, were constant across blocks. For Experiments 1a and 1b, there were 36 Test stimuli/block (18 High F0, 18 Low F0) and 12 Control stimuli/block (6 Beer, 6 Pier). In all, there were 48 trials/block and 96 trials across the experiment. Owing to its within-participant design, Experiment 1c involved half as many trials per block and the order of visual-support and no-visual-support blocks was counterbalanced. Each experiment took about 20 min to complete.

### 2.2. Experiment 1a results

Experiment 1a paired a clip art image that unambiguously differentiates /b/ versus /p/ across Exposure stimuli as additional category support, providing a conservative test of whether passive exposure to speech conveying distinct short-term regularities induces dimension-based statistical learning.

We first examined test stimulus categorization as a function of Block (Canonical, Reverse) and Test Stimulus F0 (High, Low F0) with a general linear mixed-effects regression model (GLMER). Due to the occurrence of a singular fit with the maximal random effect structure, a base model included the random intercept of Subject and a random slope of Test Stimulus (AIC = 2813.73, BIC = 2836.44). We examined fixed effects by adding predictors to the base model and observing model fit. Here, and in subsequent analyses, “Canonical” was used as the reference level for Block and “Low F0” was used as the reference level for the Test Stimulus F0. We assessed interaction effects by adding the interaction term to a model including both fixed effects.

**Fig. 2.**
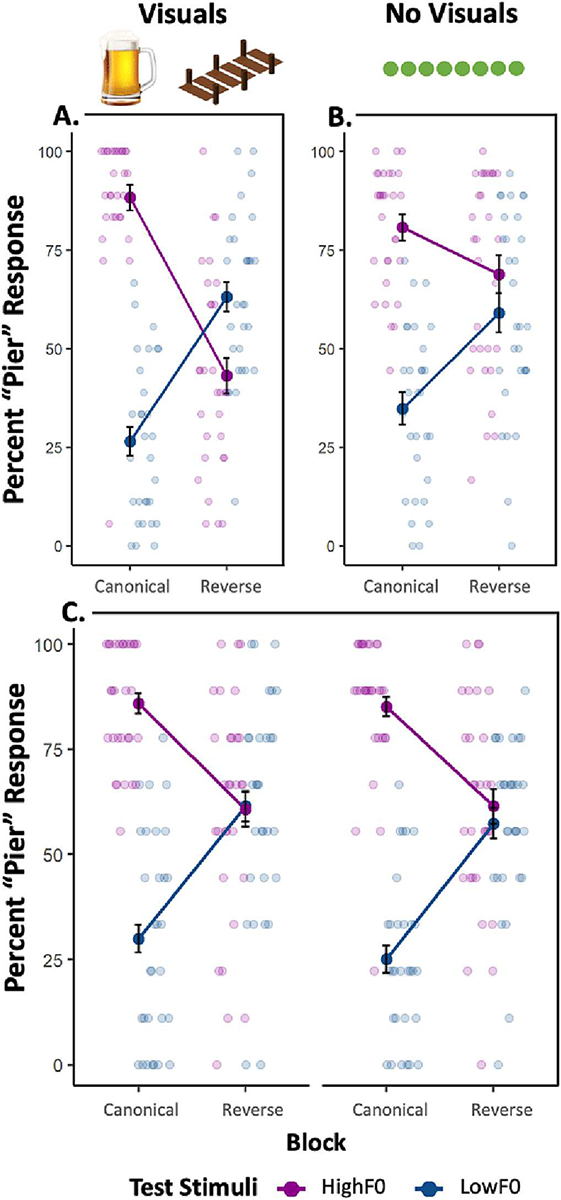
Experiment 1 Results. Mean percent “pier” responses across Canonical and Reverse blocks for High F0 (purple) and Low F0 (blue) test stimuli for the (A) Exp 1a; (B) Exp 1b; and (C) Exp 1c. Scatterplot points display individual participant’s means by block. Exposure stimuli in Exp1a were accompanied by *beer* and *pier* category support images while those in Exp1b were accompanied by a green progress bar. For Exp 1c, the left panel displays individual and group means for the “visual support” condition while the right displays that for the “no visuals” condition. Error bars represent +/−1 SE of the mean.

As shown in Fig. 2a, dimension-based statistical learning was elicited across passive exposure to the speech regularities that differentiated Canonical and Reverse blocks, as reflected by an interaction of Block and Test Stimulus F0 (β = −4.29, SE = 0.23, z = −19.04, *p* < .001, $\eta_{p}^{2}=0.85$) that significantly improved model fit (AIC = 2337.49, BIC = 2377.24, X^2^ = 452.87, p < .001) compared to an additive model that included only Block and Test Stimulus F0 fixed effects (AIC = 2788.37, BIC = 2822.43). Categorization reliant on F0 was influenced by short-term speech input regularities.

Further planned analyses inform the nature of this interaction. When distributional regularities conveyed by Exposure stimuli aligned with English VOTxF0 patterns, F0 differentially signaled *beer* and *pier*, in line with norms. Participants categorized the High F0 Test Stimulus significantly more often as *pier* than the Low F0 test stimulus (β = 3.38, SE = 0.24, z = 13.86, p < .001). When Reverse Exposure stimuli conveyed a short-term regularity that mismatched English norms, there also was a significant difference in categorization of High and Low F0 test stimuli (β = −0.91, SE = 0.21, z = −4.33, p < .001), but in the opposite direction – mirroring the reversed short-term input distribution. As we discuss below, the cross-over is unexpected and – should it replicate – may implicate passive exposure as more potent than trial-by-trial accumulation of regularities across overt categorization responses. There also were significant effects of Block (β = 1.67, SE = 0.14, z = 12.09, *p* < .001) and Test Stimulus (β = 3.38, SE = 0.24, z = 13.86, p < .001); listeners were more likely to respond *pier* in the Canonical block and to the High F0 test stimulus, across blocks.

### 2.3. Experiment 1b results

Experiment 1b eliminated category support from visual images by replacing them with neutral images of green circles. This maintained experimental details from Experiment 1a, including audio-visual temporal alignment.

Following the approach of Experiment 1a, we examined main effects and interactions in Experiment 1b. The base model accepted the maximal random effect structure of a random effect of Subject with random slopes of Block, Test Stimulus, and the Block x Test Stimulus interaction (random effects only: AIC = 2449.01, BIC = 2511.47). As in Experiment 1a, there were significant main effects of both Block (β = 1.18, SE = 0.17, z = 6.74, *p* < .001) and Test Stimulus F0 (β = 2.38, SE = 0.24, z = 10.07, *p* < .001).

As evident in Fig. 2b, the short-term Canonical and Reverse regularities impacted reliance on F0 in categorization, as evidenced by a significant interaction effect between Block and Test Stimulus F0 (β = −1.78, SE = 0.34, z = −5.19, *p* < .001, $\eta_{p}^{2}=0.49$) that significantly improved model fit (AIC = 2399.98, BIC = 2479.47, X^2^ = 18.22, *p* < .001) when compared to a model with the two main effects (AIC = 2416.19, BIC = 2490.01). Test Stimulus F0 signaled /b/ and /p/ differentially in the Canonical block (β = −2.38, SE = 0.24, z = −10.07, *p* < .001) but F0 was not effective in signaling category identity in the Reverse block (β = −0.61, SE = 0.34, z = −1.79, *p* = .074). In summary, passive exposure to short-term speech statistics is sufficient to evoke dimension-based statistical learning even without additional support from visual images that differentiate Exposure stimuli.

### 2.4. Experiment 1c results

Experiment 1c examined whether modest differences across Experiments 1a and 1b might be due to cohort differences, or whether there is a true influence of visual support. Experiment 1c was identical to Experiments 1a and 1b, except that a single sample of participants experienced trials both with and without visual support, presented in blocks counterbalanced in order. To keep the duration of the experiment the same, the number of trials in each condition was half that of Experiments 1a and 1b.

We constructed a base GLMER including the random slopes of Block (Canonical, Reverse), Test Stimulus F0 (High F0, Low F0), and their interaction varied across Subject (intercept) and iteratively added fixed effects of Visual Support (Yes, No), Block, Test Stimulus F0, and their interactions (AIC = 2484.6, BIC = 2547.0). Visual Support could not be added to the random effect structure due to singular fit. “Canonical” was used as the reference level for the Block condition, “Low F0” was used as the reference level for the Test Stimulus F0 condition, and “No Visual” was used as the reference level for visual support. Fig. 2c plots the results.

In a model with the full set of interactions, there were main effects of both Block (β = 1.56, SE = 0.25, z = 6.23, *p* < .001) and Test Stimulus F0 (β = 3.14, SE = 0.32, z = 9.84, *p* < .001), and no main effect of Visual Support (β = 0.27, SE = 0.20, z = 1.33, *p* = .185).

As in Experiments 1a and 1b, there was a Block x Test Stimulus F0 interaction (β = −2.90, SE = 0.42, z = −6.85, *p* < .001, $\eta_{p}^{2}=0.73$), consistent with the down-weighting of F0 in the Reverse block. Critically, this interaction was not modulated by Visual Support as a three-way interaction (β = −0.02, SE = 0.42, z = −0.05, *p* = .963, $\eta_{p}^{2}<0.001$), indicating that the magnitude of down-weighting did not differ with the presence of visual support.

Visual Support also did not interact with either Block (β = −0.08, SE = 0.27, z = −0.29, *p* = .769) or Test Stimulus (β = −0.21, SE = 0.32, z = −0.64, *p* = .520). Compared to a fully additive model (AIC = 2472.5, BIC = 2552.0), a model containing a Block by Test Stimulus interaction and an additive effect of Visual Support provided a significantly better fit to the data (AIC = 2440.0, BIC = 2525.2, X^2^ = 34.48, *p* < .001). This partially additive model did not significantly differ from a model of the full set of interactions (AIC = 2444.6, BIC = 2546.8, X^2^ = 1.39, *p* = .708).

In summary, three replications across 90 online participants demonstrate that passive exposure to distributions of speech input has a robust influence on the perceptual weight of acoustic dimensions in signaling speech categories. Upon encountering short-term speech regularities that depart from the language community norm in passive listening, listeners’ speech categorization reflects an adjustment of the effectiveness of the mismatching dimension in signaling category membership. The mapping from acoustic input to speech categories is dynamically tuned in response to changes in the distributional characteristics of ongoing speech input, passively experienced.

## 3. Experiment 2

How does the magnitude of dimension-based statistical learning across passive exposure compare to that observed across overt trial-by-trial categorization decisions? One possibility is that the category decisions involved in overt response paradigms amplify category activation and thereby exaggerate dimension-based statistical learning compared to well-matched passive exposure. Alternatively, experiencing distributions on a trial-by-trial basis in passive listening may be a more potent driver of perceptual weight adjustments. Experiment 2 evaluates these possibilities within a single cohort of listeners that completes both passive exposure and overt categorization tasks.

### 3.1. Method

#### 3.1.1. Participants

A group of 41 adults (30 female, 11 male) between the ages of 18 and 28 years (M = 23.77, SD = 2.84) was winnowed by 11 subjects who failed to categorize control trials with >70% accuracy to arrive at a sample of 30 participants (M = 24.07 years, SD = 2.89; 22 female, 8 male).

#### 3.1.2. Stimuli

Stimuli were identical to Experiment 1b.

#### 3.1.3. Procedure

The passive listening task was the same Experiment 1b except that there were 40 total trials total (20 trials/block) with High F0 and Low F0 Test stimuli heard 8 times per block and Control stimuli heard twice in each block. The passive task took about 10 min to complete. In the overt categorization task, each stimulus was presented in isolation, immediately followed by a black question mark prompting a *beer-pier* categorization decision by keypress. This differed from the passive listening task in which 8 exposure stimuli and a final test or control stimulus comprised a trial. Therefore, the overt categorization task had 360 total trials (180/block), with a test or control trial presented after every 8 exposure trials to align with the passive listening task. The overt categorization task took about 15 min to complete. Task order was counterbalanced across participants.

### 3.2. Results

To avoid singular fit, a base GLMER model included the random slope of Test Stimulus F0 that varied by Subject, AIC = 2296.8, BIC = 2319.1. Fixed effects and their interactions were subsequently added to the base random effects structure to assess model fit. “Canonical” was used as the reference level for Block, “Low F0” was used as the reference level for Test Stimulus F0, and “Overt” was used as the reference level for Task.

Compared to a purely additive model (AIC = 2264.5, BIC = 2303.4), a full model of interactions provided a significantly better fit (AIC = 2173.5, BIC = 2234.7, X^2^ = 98.96, *p* < .001). This model was also a significantly better fit compared to a partially additive model involving only the interaction term of Block x Test Stimulus (AIC = 2182.7, BIC = 2227.2, X^2^ = 15.17, *p* = .002). The full model with all interactions revealed main effects of both Block (β = 1.41, SE = 0.21, z = 6.66, p < .001) and Test Stimulus F0 (β = 2.96, SE = 0.33, z = 8.88, *p* < .001), as in previous experiments. There was no main effect of Task (β = 0.02, SE = 0.21, z = 0.11, *p* = .917), indicating that the overall proportion of *pier* responses did not differ significantly for overt versus passive tasks. The effect of Task significantly interacted with Block (β = −0.89, SE = 0.29, z = −3.04, *p* = .002) and with Test Stimulus F0 (β = −0.80, SE = 0.34, z = −2.36, *p* = .018).

**Fig. 3.**
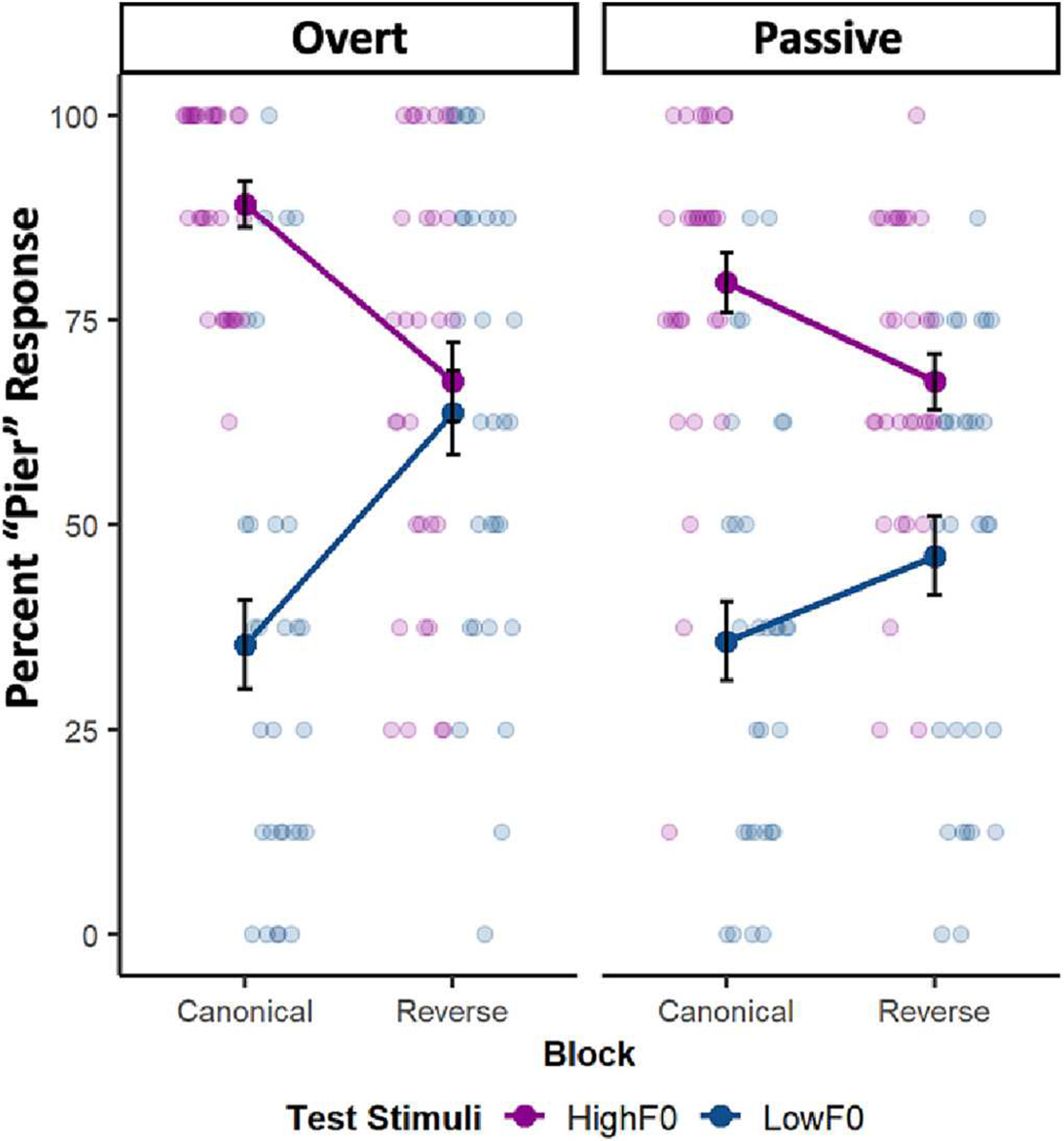
Experiment 2 Results. Mean percent “pier” response as a function of short-term speech input regularities. Light symbols show individual means and dark symbols show group means (error bars represent ±1 SE) across High F0 and Low F0 Test stimuli in Canonical and Reverse blocks for (A) overt category decisions and (B) passive listening.

As Fig. 3 shows, there was a significant interaction of Block and Test Stimulus F0 (β = −2.85, SE = 0.33, z = −8.63, *p* < .001, $\eta_{p}^{2}=0.72$), the hallmark of dimension-based statistical learning, across tasks. Considering each task separately, the interaction was significant for both the overt categorization task (β = −3.06, SE = 0.35, z = −8.80, *p* < .001, $\eta_{p}^{2}=0.75$) and the passive listening task (β = −1.16, SE = 0.29, z = −3.94, *p* = .002, $\eta_{p}^{2}=0.29$).

This was moderated by Task in a three-way interaction (β = 1.67, SE = 0.44, z = 3.76, *p* < .001, $\eta_{p}^{2}=0.47$). Overall, with data pooled across the counterbalanced orders, F0 was down-weighted less in the passive listening task than in the overt categorization task. This is consistent with the possibility that overt category decisions and responses amplify the effects of dimension-based statistical learning, perhaps by exaggerating category activation effects. Yet, we note that the potential for carry-over effects across the task manipulations warrants caution. When the passive paradigm preceded the overt task, there was no significant difference in the magnitude of F0 down-weighting (Task type x Block x Test Stimulus, β = 0.08, SE = 0.63, z = 0.13, *p* = .898) observed across passive and overt paradigms. However, when participants first made explicit category decisions and then experienced passive exposure, we observed significantly less down-weighting of F0 perceptual weight across passive exposure to the accent (Task type x Block x Test Stimulus, β = 3.48, SE = 0.69, z = 5.07, *p* < .001). In summary, participants track short-term speech regularities across individual trials in the overt task and mere exposure in the passive task, with the accent introduced by the Reverse block diminishing the perceptual weight of F0 in speech categorization. There are carry-over effects of task that might be examined in future research.

## 4. Experiment 3

Experiment 3 examines the time course of dimension-based statistical learning across passive exposure by asking whether listeners track trial-wise short-term regularities when opposing short-term regularities are mixed in presentation.

### 4.1. Methods

#### 4.1.1. Participants

A total of 36 younger adults (16 male, 20 female) ages 18 to 30 years (M = 25.36, SD = 3.5) were recruited. Six participants were excluded from further analyses for failing to reach 70% accuracy on control trials, for a total of 30 participants included in subsequent analyses.

#### 4.1.2. Stimuli and procedure

Stimuli, experimental design, and procedure mirrored Experiment 1b, except that Canonical and Reverse trials were mixed instead of blocked across trials. This mixing did not change the total number of trials (96 total trials). Each subject heard a unique trial order.

### 4.2. Results

**Fig. 4.**
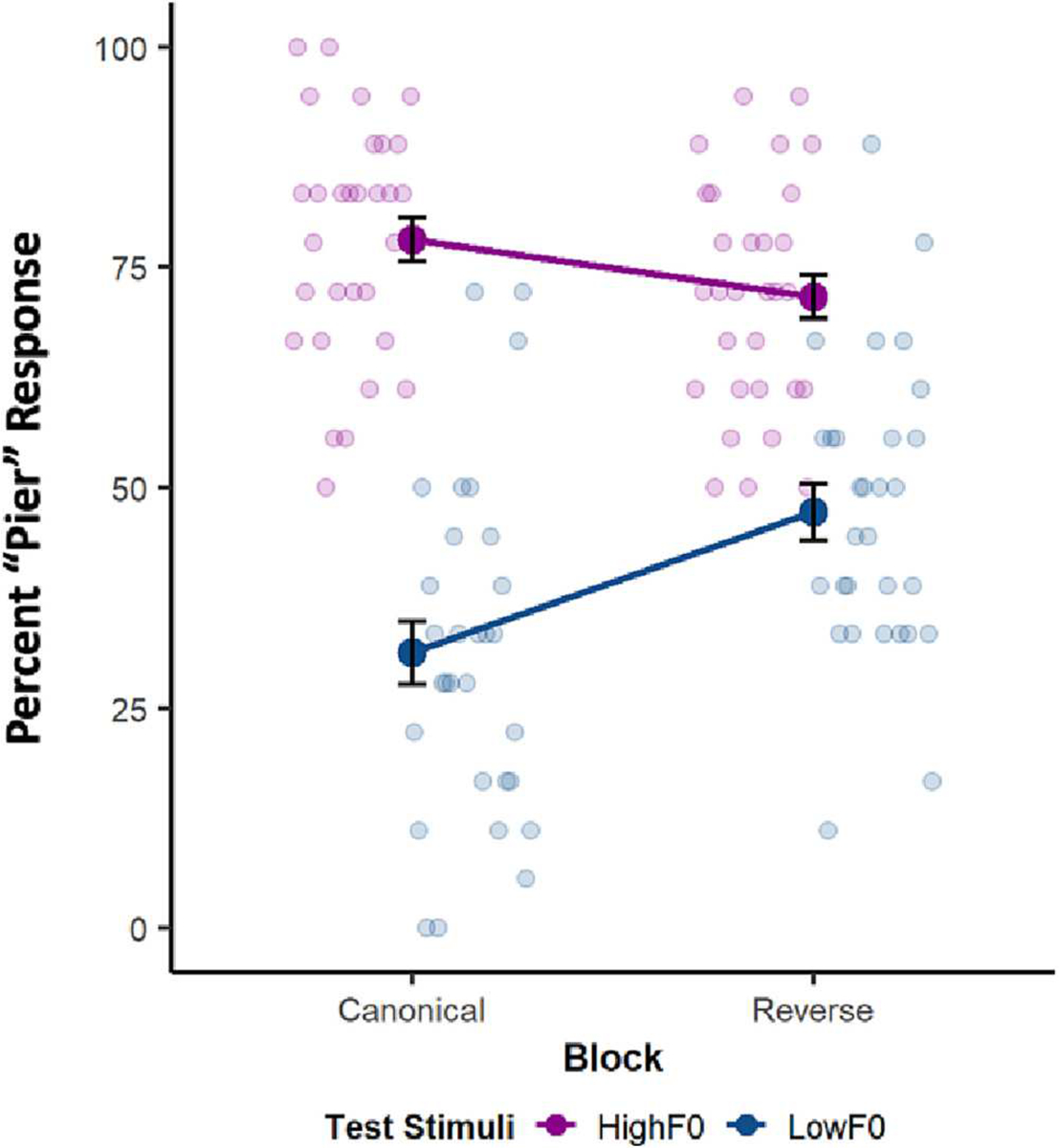
Experiment 3 Results. Mean percent “pier” response as a function of the short-term regularity conveyed across the Exposure sequence High F0 (purple) and Low F0 (blue) Test stimuli. Light symbols show individual means and dark symbols show group means (error bars represent ±1 SE).

The base GLMER model included a random slope of Test Stimulus F0 (High F0, Low F0) that varied by Subject, (AIC = 2635.6, BIC = 2658.3). Fixed effects of Regularity (Canonical, Reverse) and Test Stimulus and their interaction were then added to this base model. Fig. 4 shows the results.

Dimension-based statistical learning was evident across passive exposure even when Canonical and Reverse short-term regularities were mixed on a trial-wise basis, as evident in a significant interaction of Regularity and Test Stimulus F0 (β = −1.11, SE = 0.19, z = −5.65, *p* < .001, $\eta_{p}^{2}=0.28$). Dimension-based statistical learning operated across as few as 8 stimuli conveying a short-term distributional regularity and in the context of rapidly changing distributions. Compared to an additive model (AIC = 2595.7, BIC = 2629.8), addition of a Regularity x Test Stimulus F0 interaction term significantly improved model fit (AIC = 2565.6, BIC = 2605.3, X^2^ = 32.11, *p* < .001), further supporting the presence of F0 down-weighting in the context of Reverse compared to the Canonical speech regularities experienced across passive exposure. Like all previous experiments, there were significant main effects of both Regularity (β = 0.75, SE = 0.13, z = 5.59, *p* < .001) and Stimulus Type (β = 2.21, SE = 0.22, z = 10.01, *p* < .001). In a sense, mixed presentation of Canonical and Reverse short-term regularities across trials simulates an unreliable speaker who rapidly shifts accent. Listeners are sensitive to these regularities in passive listening, as evidenced by a re-weighting of the influence of F0 in speech categorization.

## 5. Discussion

Listeners track distributional regularities across passive exposure to speech even when they do not make overt categorization judgements. Upon encountering short-term distributional regularities that depart from language norms, as in accented speech, listeners rapidly adjust the perceptual weight, or effectiveness, with which incoming acoustic dimensions signal speech categories. Observing this dimension-based statistical learning across passive exposure to short-term distributional regularities opens the possibility that dimension-based statistical learning may play a role in natural listening contexts, such as daily conversation and listening to spoken media. It also demonstrates that explicit speech categorization decisions are not necessary to trigger perceptual re-weighting based on input statistics. These conclusions are supported across five independent samples (*N* = 180 total participants) drawn from diverse young adults recruited and tested online. Indeed, results across the three replications of Experiment 1 demonstrate that passive exposure to speech regularities is sufficient to drive dimension-based statistical learning, without the need for additional support for category activation from visual images. Experiments 2 and 3 provide further converging evidence. Dimension-based statistical learning persists even in the volatile statistical context of Experiment 3, in which regularities could shift trial-by-trial.

One might question whether this new paradigm is entirely “passive” in that listeners report a category decision for test stimuli. In this regard, it is important to note that these explicitly labeled test stimuli were constant across conditions. Learning across the distinct speech regularities happens across the sequence of sounds that precedes test stimuli and therefore must play out over passive exposure. Statistical learning experiments utilizing passive exposure often expose listeners to long passages of input and then introduce an active behavioral task to assess learning. Here, given the rapid nature of dimension-based statistical learning, we are able to take this approach at the trial level.

As described above, dimension-based statistical learning appears to be dependent upon speech category activation driven by a disambiguating information source, such as an unambiguous acoustic dimension (like VOT in the present studies; Wu and Holt, 2022) or top-down resolution through lexical context (Zhang et al., 2021). This activation may allow for predictions of typical patterns of multidimensional speech input, and error-based adjustments when input mismatches expectations. Yet, statistical learning across passive exposure has been more typically considered as unsupervised learning (Fiser and Aslin, 2001; Saffran et al., 1996). Observing dimension-based statistical learning across passive exposure, as we do here, suggests another possibility that departs from traditional accounts of statistical learning in two important ways.

First, the present results underscore the importance of language-specific knowledge, acquired over the long-term, in resolving ambiguity and driving learning. When distributional input conveys information sufficient to differentially activate speech categories (as here via the unambiguous VOTs of passively experienced exposure stimuli), then learning may operate via the predictions generated by category activation, and error signals arising when input does not match predictions. In this way, the disambiguating dimensions present in multidimensional speech input can serve as ‘teacher signals’ by activating internal category representations that produce both predictions and errors to drive learning. Indeed, even beyond dimension-based statistical learning, other forms of passive statistical learning that have been thought to emerge from unsupervised learning may, in fact, get a boost from internal predictions if perceptual input aligns well enough with prior experience to activate existing representations.

Second, the present results do not implicate a change in category representation. It would be disadvantageous to overwrite or distort native category representations after brief encounters with input that deviates from these norms. Instead, dimension-based statistical learning appears to adjust the *effectiveness* of acoustic dimensions in signaling speech categories. This can be accommodated through transient adjustments to the connection-weights (efficiency) of communication from acoustic input to speech categories. This generates rapid and *short-term* flexibility, without producing rapid changes to long-term category representations that could lead to perceptual instability. Lehet and Holt (2020) lend support for this possibility by demonstrating that down-weighting of an acoustic dimension in signaling speech categories, as in dimension-based statistical learning, does not diminish the effectiveness of the dimension – conveyed by the *same* speech sound – in evoking lower-level, pre-categorical context-dependent interactions with other speech sounds. In this way acoustic input dimensions persist as a potent driver of pre-categorical interactions even when their influence in differentially signaling speech categories may diminish. A model like this has the advantage of reconciling the classic stability versus plasticity dilemma faced by systems that need to represent long-term statistical regularities while also maintaining flexibly adjust to short-term deviations.

Of course, all prior studies of dimension-based statistical learning have required explicit categorization decisions. Thus, the best evidence for error-driven learning is from tasks demanding explicit categorization decisions and a trial-by-trial accumulation of distributional statistics (Wu and Holt, 2022; Zhang et al., 2021; Lehet and Holt, 2020). Yet, the present results make clear that the pattern of dimension down-weighting associated with dimension-based statistical learning emerges with passive exposure to short-term input distributions that deviate from English norms. Explicit decisions are not necessary for dimension-based statistical learning; passive exposure is sufficient.

Experiment 2 provided a direct comparison of overt-response and passive-listening paradigms among the same listeners. The greater down-weighting observed in the overt categorization compared to the passive exposure task is consistent with the possibility that overt category decisions and responses amplify the effects of dimension-based statistical learning. Yet, this evidence is equivocal, as task order also impacted outcomes. Experiment 2 provides a clear demonstration that – under the right conditions of task order – short-term regularities that build up across many trials involving explicit category decisions and passive exposure to the same distributions in a trial-wise manner produce comparable dimension-based statistical learning. We leave it to future work to understand how the substantial task differences in the presence of trial-wise categorization decisions, the time course of the build-up of distributions (across vs. within trials) and, presumably, active engagement or attention impact the nature and degree of dimension re-weighting. Paradigms like this one open this possibility in studies of statistical learning because of the trial-by-trial evaluation of input statistics on the perceptual weighting of input dimensions.

Finally, Experiment 3 assessed the degree to which dimension-based statistical learning elicited through passive listening is robust to trial-by-trial variability in short-term regularities, and whether the adjustment of perceptual weights requires build-up of statistical information across many exposures. The statistically volatile context created by shifting randomly between Canonical and Reverse distributions on a trial-by-trial basis led to significant dimension-based statistical learning, although the effect size was smaller than the more consistent contexts.

The preservation of perceptual flexibility in a highly variable speech environment additionally contributes support to dimension-based statistical learning as a learning mechanism valuable in in ecologically relevant contexts, like adapting rapidly across talkers in a group conversation.

## 6. Conclusion

In sum, the present study offers five replications of dimension-based statistical learning elicited through passive exposure to short-term distributions in speech input. In conjunction with previous work, the present data support the proposition that error-driven learning—whereby activation of internal speech category representations provides teaching signals that generate predictions—can drive statistical learning even across passive exposure.

Ultimately, this work encourages further theoretical discourse. When structured inputs consistently activate established internal category representations, discrepancies between canonical categorical predictions and experienced input-output regularities can drive statistical learning, and thus need not operate solely via an unsupervised process.

## Figures and Tables

**Table 1:** Participant demographic information for Experiments 1a–c.

	Before exclusion				After exclusion			
Exp	N	Age Mean (SD)	Age range	Gender (F,M,Other)	N	Age Mean (SD)	Age range	Gender (F,M,Other)
Exp 1a	38	23.53 (3.48)	19–31	21, 15, 2	30	23.8 (3.40)	19–30	16, 12, 2
Exp 1b	34	22.71 (3.29)	18–29	16, 18	30	22.33 (3.09)	18–29	13, 17
Exp 1c	37	24.55 (3.60)	18–31	30, 6, 1	30	24.2 (3.63)	18–30	24, 5, 1

**Table 2:** GLMER model outputs.

	Effect	Estimate	Std. Error	z value	Pr(>|z|)	Sig.
Exp 1a	(Intercept)	−1.10	0.16	−6.85	0.000	***
	Block	1.67	0.14	12.09	0.000	***
	Test Stimulus	3.38	0.24	13.86	0.000	***
	Block x Test Stimulus	−4.29	0.23	−19.04	0.000	***
Exp 1b	(Intercept)	−0.74	0.19	−3.86	0.000	***
	Block	1.18	0.17	6.74	0.000	***
	Test Stimulus	2.38	0.24	10.07	0.000	***
	Block x Test Stimulus	−1.78	0.34	−5.19	0.000	***
Exp 1c	(Intercept)	−1.25	0.22	−5.73	0.000	***
	Visual Support	0.27	0.20	1.33	0.185	
	Block	1.56	0.25	6.24	0.000	***
	Test Stimulus	3.14	0.32	9.84	0.000	***
	Visual Support x Block	−0.08	0.27	−0.29	0.769	
	Visual Support x Test Stimulus	−0.21	0.32	−0.64	0.520	
	Block x Test Stimulus	−2.90	0.42	−6.85	0.000	***
	Visual Support x Block x Test Stimulus	−0.02	0.42	−0.05	0.963	
Exp 2	(Intercept)	−0.73	0.24	−3.09	0.002	**
	Task Type	0.02	0.21	0.10	0.917	
	Block	1.41	0.21	6.66	0.000	***
	Test Stimulus	2.96	0.33	8.88	0.000	***
	Task Type x Block	−0.89	0.29	−3.04	0.002	***
	Task Type x Test Stimulus	−0.80	0.34	−2.36	0.018	*
	Block x Test Stimulus	−2.85	0.33	−8.63	0.000	***
	Task Type x Block x Test Stimulus	1.67	0.44	3.76	0.000	***
Exp 2 (Passive first)	(Intercept)	−1.04	0.29	−3.60	0.000	***
	Task Type	0.34	0.29	1.17	0.241	
	Block	1.15	0.29	3.97	0.000	***
	Test Stimulus	2.86	0.46	6.19	0.000	***
	Task Type x Block	−0.49	0.40	−1.23	0.218	
	Task Type x Test Stimulus	0.03	0.48	0.06	0.955	
	Block x Test Stimulus	−2.08	0.44	−4.74	0.000	***
	Task Type x Block x Test Stimulus	0.08	0.63	0.13	0.898	
Exp 2 (Overt first)	(Intercept)	−0.43	0.40	−1.09	0.275	
	Task Type	−0.32	0.30	−1.06	0.292	
	Block	1.86	0.33	5.58	0.000	***
	Test Stimulus	3.30	0.53	6.19	0.000	***
	Task Type x Block	−1.50	0.45	−3.37	0.001	**
	Task Type x Test Stimulus	−1.62	0.53	−3.04	0.002	**
	Block x Test Stimulus	−4.04	0.55	−7.34	0.000	***
	Task Type x Block x Test Stimulus	3.48	0.69	5.07	0.000	***
Exp 2 (Passive Only)	(Intercept)	−0.70	0.23	−3.07	0.002	**
	Block	0.51	0.20	2.52	0.012	*
	Test Stimulus	2.12	0.30	7.13	0.000	***
	Block x Test Stimulus	−1.16	0.30	−3.94	0.000	***
Exp 2 (Overt Only)	(Intercept)	−0.79	0.28	−2.78	0.005	**
	Block	1.53	0.23	6.74	0.000	***
	Test Stimulus	3.17	0.38	8.26	0.000	***
	Block x Test Stimulus	−3.06	0.35	−8.80	0.000	***
Exp 3	(Intercept)	−0.87	0.16	−5.38	0.000	***
	Block (Regularity)	0.75	0.13	5.59	0.000	***
	Test Stimulus	2.21	0.22	10.02	0.000	***
	Block (Regularity) x Test Stimulus	−1.11	0.20	−5.65	0.000	***

* <0.05,

** < 0.01,

*** < 0.001.

**Table 3:** GLMER model parameters.

	AIC	BIC	logLik	deviance	df.resid
Exp1a Formula	2337.49	2377.24	−1161.75	2323.49	2153
	*Correct ~ (Test Stimulus|Subject)* + Block*Test Stimulus				
Exp1b Formula	2399.98	2479.47	−1185.99	2371.98	2146
	Correct ~ (Block*Test Stimulus|Subject) + Block*Test Stimulus				
Exp1c Formula	2444.63	2546.83	−1204.32	2408.63	2142
	Correct ~ (Block*Test Stimulus|Subject) + Visuals*Block*Test Stimulus				
Exp2 Formula	2173.54	2234.7	−1075.77	2151.54	1909
	*Correct ~ (Test Stimulus|Subject)* + Task*Block*Test Stimulus				
Exp2 (Passive first) Formula	1096.9	1150.5	−537.5	1074.9	949
	*Correct ~ (Test Stimulus|Subject)* + Task*Block*Test Stimulus				
Exp2 (Overt first) Formula	1054.9	1108.4	−516.4	1032.9	949
	*Correct ~ (Test Stimulus|Subject)* + Task*Block*Test Stimulus				
Exp2 (Passive only) Formula	1163.12	1197.19	−574.56	1149.12	953
	*Correct ~ (Test Stimulus|Subject)* + Block*Test Stimulus				
Exp2 (Overt only) Formula	1020.58	1054.65	−503.29	1006.58	953
	*Correct ~ (Test Stimulus|Subject)* + Block*Test Stimulus				
Exp3 Formula	2565.59	2605.34	−1275.8	2551.59	2153
	*Correct ~ (Test Stimulus|Subject)* + Block (Regularity)*Test Stimulus				

## Data Availability

The experiment reported in this article was not formally preregistered. The data and the stimulus set have been made available on a permanent third-party archive: https://osf.io/87skr/
